# Phylogenetic signal and functional categories in Proteobacteria genomes

**DOI:** 10.1186/1471-2148-7-S1-S7

**Published:** 2007-02-08

**Authors:** Iñaki Comas, Andrés Moya, Fernando González-Candelas

**Affiliations:** 1 Instituto Cavanilles de Biodiversidad y Biología Evolutiva. Universidad de Valencia. Apartado Oficial 22085, Valencia E-46071, Spain

## Abstract

**Background:**

A comprehensive evolutionary analysis of bacterial genomes implies to identify the hallmark of vertical and non-vertical signals and to discriminate them from the presence of mere phylogenetic noise. In this report we have addressed the impact of factors like the universal distribution of the genes, their essentiality or their functional role in the cell on the inference of vertical signal through phylogenomic methods.

**Results:**

We have established that supermatrices derived from data sets composed mainly by genes suspected to be essential for bacterial cellular life perform better on the recovery of vertical signal than those composed by widely distributed genes. In addition, we show that the "Transcription" category of genes seems to harbor a better vertical signal than other functional categories. Moreover, the "Poorly characterized" category performs better than other categories related with metabolism or cellular processes.

**Conclusion:**

From these results we conclude that different data sets allow addressing different questions in phylogenomic analyses. The vertical signal seems to be more present in essential genes although these also include a significant degree of incongruence. From a functional perspective, as expected, informational genes perform better than operational ones but we have also shown the surprising behavior of poorly annotated genes, which points to their importance in the genome evolution of bacteria.

## Background

The genomes of bacteria harbor different evolutionary signals as the result of the different evolutionary processes that act upon them. As a consequence, the information encoded in these genomes can be divided into three main categories: vertical signals, non-vertical signals and phylogenetic noise. The reconstruction of bacterial evolution and the appraisal of the different forces that have shaped their genomes depend on the disentangling of these signals.

The vertical signal is associated to the transmission of genetic information from ancestors to descendants. From a genomic perspective, this signal resides in the set of true orthologs shared by microbial genomes. The non-vertical signal arises as the result of evolutionary processes that do not involve the immediate ancestors as donors of genetic material. The two most common processes at a genome scale level originating this signal are duplications and horizontal gene transfers. Paralogs are those genes resulting from a process of duplication. After their origin, paralogs may have different fates from neo or sub-functionalization to extinction through gene disintegration [1]. Xenologs are genes horizontally transmitted from a non-relative of the recipient genome [2,3]. The existence of horizontal gene transfer among microorganisms is known from quite long ago [4] and is currently recognized as one of the main processes influencing the evolution of bacteria [5,6]. The term synologs denotes the presence of more than one homolog within a genome regardless of the origin of the duplicate copies (paralogy or xenology) [7]. Lastly, phylogenetic noise could have different sources and embrace cases of insufficient phylogenetic signal or complex evolutionary patterns that limit phylogenetic inference [8].

In principle, it could be expected that the largest group of genes in bacteria belong to the vertical category [9,10]. Most of the genome is vertically inherited every generation, although the most important innovations seem to be acquired as the result of horizontal transfer events [11] and, to a lesser degree, of duplications [12]. However, the exact fraction of genes belonging to each category is variable among different groups, even species, and difficult to assess. In fact, there is disagreement about the extent to which non-vertical processes, mainly lateral gene transfers, influence the inference of genome phylogenies and the existence of a species tree for bacteria. If the rate of lateral gene transfer is high, then a phylogeny that relies on ancestor-descendant relationships will not be able to reflect the evolution of bacterial genomes that might be described better by means of networks [13]. However, if this rate is low enough then we will be able to represent bacterial evolution as a tree and not as a network [14]. In their extreme version, these two positions deny the importance of the vertical or the non-vertical signals, respectively. Only those studies capable of reporting both signals and measuring the possible influence of phylogenetic noise will be addressing properly the evolution of bacterial genomes.

In traditional phylogenetic analysis different molecules have been proposed to be good, reliable markers of bacterial evolution. However, the most widely used method has been the analysis of 16S rDNA, which was demonstrated to contain a good vertical signal and able to recover accurate phylogenies at different phylogenetic levels [15,16]. Currently, this is still the most widely used tool in bacterial taxonomy [17]. However, the availability of a growing number of complete bacterial genomes is confirming the necessity of verification of 16S rDNA results with information encoded in protein coding genes [18]. Firstly, because the evolutionary scenario derived from gene trees is often incongruent with that of 16S rDNA and, secondly, because the evolution of one gene does not necessarily reflect the vertical signal of the whole genome. Current works are taking advantage of the relatively recent development of phylogenomic methodologies [19-21]. Two of the most common approaches are the supermatrix and supertrees analyses. A supermatrix is created by the concatenation of multiple partitions, usually genes in this phylogenomic context. It has the potential of adding up the individual phylogenetic signals with the aim of recovering the main one. The supertree approach uses an alternative route: instead of analyzing all the partitions in a single analysis it recovers the gene trees of the individual genes and generates a tree (supertree) [22,23]. This supertree is a summary of the underlying source trees and it is supposed to be the most compatible topology for all of them.

On the other hand, not only the phylogenomic methodology is important but also the data set to which it is applied is of relevance. The nature of the genes that compose the data set to be analyzed can have a direct incidence on the phylogeny recovered and on the phylogenetic signals contained therein [24]. From any genome, which is composed by a mixture of signals, different subsets can be derived. The term 'minimal genome' has been used to describe the set of genes that are supposed to be essential for a self-sustainable cell live [25]. There is no single, unique minimal genome and several proposals have been put forward [25,26]. However, a recent review of different approaches has proposed a synthesis of 206 genes as the minimal genome needed for cellular life [27]. It is expected that these genes, most of them characterized by their essentiality and their central role in the metabolic network, encode a good, vertical signal in agreement with the complexity hypothesis [28,29].

Nevertheless, essentiality is not the only factor that could influence the presence of vertical signal in a set of genes. It is also important that these genes are shared by all the taxa analyzed due to restrictions in the applicability of some phylogenomic methods [30]. Consequently, a core of genes suitable for the phylogenomic analysis can be defined by the universality of their presence in all the genomes considered. The universality of this core is, in consequence, another factor to consider in the analysis of the evolutionary vertical signal of bacterial genomes.

In this work, we have centered on how to identify and extract the vertical signal from a real data set of bacterial genomes in the presence of incongruence. We have performed experiments to analyze the performance of two phylogenomic methods, supermatrix and supertrees, on the inference of vertical signals. We have chosen 21 Proteobacteria genomes and have worked with the corresponding putative orthologs of the 579 protein coding genes of *Blochmannia floridanus*, a γ-Proteobacteria endosymbiont of carpenter ants [31]. In a previous work (Comas et al., submitted) we derived a reference tree (RT) for these genomes, which was supposed to grasp the vertical relationship among the species. This tree allowed us to test the presence of incongruence by comparing the RT to each gene tree. In this context, by incongruence we mean the presence of non-vertical signals or phylogenetic noise in the set of genes to be used in phylogenetic/phylogenomic analysis although how to address the source(s) of such incongruence is out of the scope of this paper. However, we study the effect of the presence of incongruence in the performance of the two phylogenomic methodologies mentioned above and address several points about the phylogenetic signal contained in the different functional categories and the role of essentiality and universality in the correct inference of vertical evolution.

## Results

The first step in a phylogenomic analysis is to obtain a reliable data set of putative orthologs for the genomes being considered. In this case, we searched for putative orthologs of the 579 protein coding genes of the *Blochmannia floridanus *genome in 20 additional Proteobacteria genomes (Table 1). The search identified 200 protein coding common genes which composed what we called the 'universal' core, thus characterized by (quasi)universal genes. Of these, 133 genes were coincident with the proposal of a minimum number of genes for a self-sustainable cell by Gil et al. [27] and composed what we called the 'essential' core, whose genes not only are universally distributed but also suspected to have an essential functional role. The distinction is important because minimal genome proposals take into account not only essential genes but also genes whose function could be replaced by other, alternative genes not included in the proposal. However, those genes included in 'minimal genome' proposals which have a universal distribution are probably essential genes.

**Figure 1:**
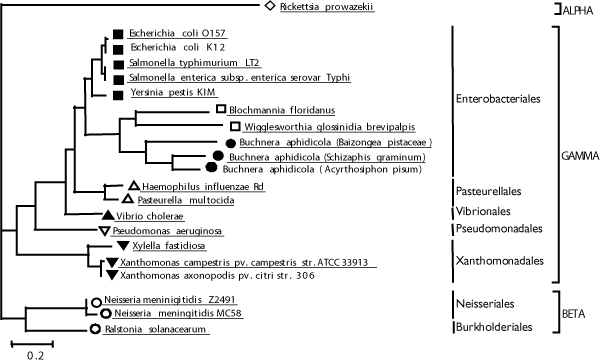
**Supermatrix analyses**. Number of concatenates out of 100 that recovered the RT (columns, left y-axis) for the 'essential' (blue) and the 'universal' (green) cores. The lines represent the average Robinson-Foulds distance (right y-axis) with standard errors from the 100 concatenates that compose each category for the 'essential' (blue) and 'universal' (green) cores.

Our first approximation to the problem of analyzing the vertical signal of these genomes consisted in comparing the performance of the 'universal' and 'essential' cores in a supermatrix analysis. We generated 100 random concatenates of 10, 20, 30, 40, 50 and 60 genes for each core and analyzed their corresponding phylogenetic trees. Figure 1 summarizes the results of two metrics to evaluate the efficiency of each data set in recovering a reference tree (RT) congruent with current taxonomical classification of the species analyzed.

The 'essential' core performed better than the 'universal' core. The 'essential' core recovered the reference tree in all 60-genes concatenates generated, whereas the 'universal' core with 60 genes concatenated only yielded a null Robinson-Foulds (RF) distance to the reference tree in 41 of the 100 concatenates. In addition, the mean topological distance reflected the differences between the two data sets. The average initial topological distances were 3.56 and 2.62 for the 'universal' and the 'essential' core concatenates, respectively. The behavior of the distance metric when the number of genes in the concatenates increased from 10 to 60 genes reflected very different dynamics for the two core sets. While the 'essential' core concatenates reduced the distance to the RT as more genes were added, the 'universal' core increased the gap as more genes were incorporated in the concatenates. The final value obtained for the 60-genes concatenates reflected this clear discrepancy: concatenates for the 'essential' core had RF distances of zero, since all of them recovered the reference tree, while the average distance of 60-genes concatenates from the 'universal' core was 5.78. The difference in the performance between these two data sets must reside, at least to a certain extent, in the 67 genes present in the 'universal' core and absent from the 'essential' core. In consequence, we included this subset of 67 genes in subsequent analyses and denoted it as 'non-essential' core.

When the complete sets of genes in the 'universal' and 'essential' cores were used to obtain the corresponding concatenates, the maximum likelihood trees showed identical topology to the reference tree (RF distance = 0). The same analysis with the 'non-essential' core resulted in a topology with RF distance = 4 to the reference tree, due to the unresolved position of Xanthomonadales at the base of the tree (not shown).

**Figure 2:**
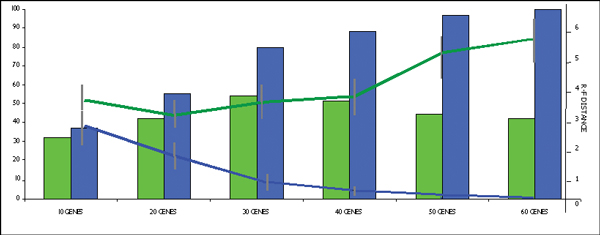
**Percentage of genes in each functional category**. Colors specify the '*Blochmannia*' (red), the 'essential' (green) and the 'universal' (blue) cores.

Once the overall phylogenetic signal in the 'universal' and 'essential' cores had been evaluated, we proceeded to study the relationship between functional assignment of the genes and performance of the phylogenomics methods described. Table 2 shows the description of each functional category whereas Figure 2 shows the contribution in percentage of each category to each data set. As expected, both the 'universal' and 'essential' cores had an enriched fraction of the informational categories while other categories had almost disappeared. In this analysis we were interested in comparing the 'universal' and the 'essential' core and also the 'Blochmannia' core, for which we had to introduce a supertree analysis, since in the latter the unequal number of sequences in the 579 multiple alignments prevented the application of a concatenate analysis. Also, due to the small number of genes present in the 'non-essential' core in the different functional categories considered, we did not include this subset in this analysis.

**Figure 3:**
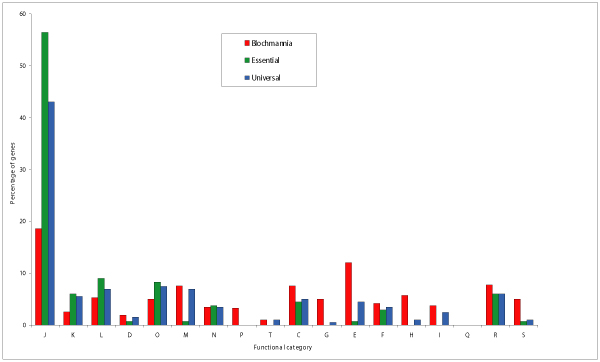
**Supermatrix and supertree functional analyses**. The categories recovering the reference tree through supermatrix (green), supertree (blue) or both methods (red) are shown as filled columns. For each category, the first column represents the results obtained with the '*Blochmannia*' core, the second column corresponds to the 'essential' core and the third column to the 'universal' core. The height of each column represents the number of genes in each functional category for the three data sets.

**Figure 4:**
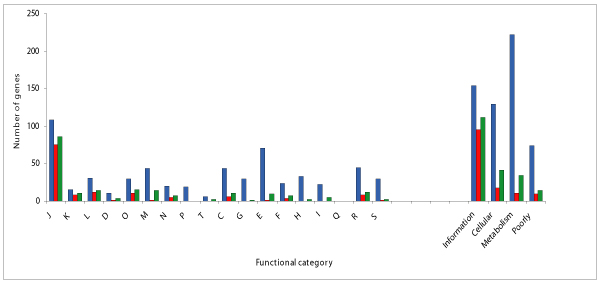
**Comparisons (RF distances) between concatenate trees, by functional category, and the reference tree**. The phylogenies for concatenates of genes in the 'essential' and 'universal' cores and functional category were derived by maximum likelihood. Cases marked with an asterisk indicate that no genes were present in the corresponding category for the 'essential' core.

A summary of the supertree and concatenate analyses is shown in Figure 3. Overall, the K ('Transcription') and the J ('Translation') categories, both related to information processes, presented the best vertical signal. For the transcription category both supermatrix and supertree approaches recovered the RT of the 'universal' and 'essential' cores as did the supertree method when applied to the 'Blochmannia' core subset. The reference tree was recovered from the subset of genes in the 'Translation' category only in the supermatrix analysis for the 'universal' and 'essential' cores, but neither in the supertree nor in the 'Blochmannia' core. The other informational category, related to replication (L), did not recover the RT in any case. The supertree derived from all the individual trees of informational genes always recovered the RT as shown in Figure 4. In the remaining categories, the RT was obtained only in a few cases. For the general categories, only the 'Blochmannia' core subset of 'Cellular processes' recovered the RT in the supertree analysis. Among the additional specific functional categories, only genes related to posttranslational modification (category O), like chaperones, seemed to retain a good vertical signal. However, two cases grabbed our attention: on the one hand, the two concatenates derived from the 'Cell motility and secretion' (N) category recovered the RT; on the other hand, the general function (R) category also behaved well in the concatenate analysis.

For a more detailed quantitative analysis, we also analyzed the topological distance of the concatenate trees derived from each of these categories to the RT. Figure 4 shows the distances from the maximum likelihood-based phylogenies obtained with the concatenates derived from the 'universal' and 'essential' cores. The general category with the shortest distances to the RT was that of informational genes whereas the others had higher distances, above all the metabolism category. Surprisingly, the second category with shortest distance to the RT was that of 'poorly characterized' genes which comprises those of 'General function' (R) and 'Unknown function' (S). In fact, a detailed analysis of the more specific categories showed that the R category was the main contributor to the short distance of the general category, recovering the RT tree in both data sets. Meanwhile, categories G ('Carbohydrate transport and metabolism') and T ('Signal transduction mechanisms') presented the largest distances among specific categories. On the other side, categories O and N that were identified with good vertical signal were the two categories, apart from the informational, with shortest distances with respect to the RT.

Finally, we analyzed the performance of the individual gene trees in the different data sets for recovering the reference tree topology. The results were very similar for the 'universal', the 'essential' and the 'non-essential' cores, with average RF distance values of 12.19, 12.00 and 12.57, respectively. This statistic was not computable for the 'Blochmannia' core as the number of sequences varies among the 579 individual gene trees considered. The results of the SH tests, at α = 0.05, for each gene tree revealed a rejection rate of 29.5%, 29%, 27% and 34.3% for the '*Blochmannia*', the 'universal', the 'essential' and the 'non-essential' cores, respectively (Table 2). The same analyses were carried out taking into account the functional assignment of the genes. Only those genes of the K ('transcription') category present in the 'universal' and 'essential' core data sets showed a significantly lower rejection rate than the mean of their corresponding data sets. Conversely, genes from the 'non-essential' core in the E ('Amino acid transport and metabolism') and I ('Lipid metabolism') categories had a significantly higher rejection rate of the RT using the SH test (Table 2).

## Discussion

One of the main questions in phylogenomic analyses based on sequence information is the composition of the data set used. We have generated three different data sets derived from the genes present in the endosymbiont *Blochmannia floridanus *and other 20 genomes. These data sets, denoted '*Blochmannia*' core, 'universal' core and 'essential' core, have allowed us to study the influence of different, presumably important factors on bacterial phylogenomics.

The main question we wanted to address was whether essentiality and universality were important factors influencing the efficiency of the commonly used concatenate methodology. Genes common to the 21 genomes, therefore expected to be quasi-universal at least at the Proteobacteria taxonomic level, were included in the 200-gene data set thus conforming the 'universal' core. On the other hand, the 133-genes common to the 21 genomes and simultaneously proposed to be minimal for a self-sustainable life conformed the 'essential' core, whose most relevant feature is essentiality. Their performance in the concatenate analyses was completely different: the 'essential' core recovered the RT with fewer genes and with higher frequency than the 'universal' core. Clearly, essentiality seems to be an important factor. In fact, while the addition of genes had little effect over the 'universal' core, in the 'essential' core the mean distances to the RT reduced continuously until becoming null when 60 genes were concatenated. These results indicate that although the vertical signal is strong in the 'universal' core it still includes incongruent genes and therefore universality does not necessarily mean absence of factors like phylogenetic noise or lateral gene transfer [32]. Meanwhile, 'essential' genes seem to have an even stronger vertical signal, a result expected because of the increased proportion of informational genes in the 'essential' core data set [29,33]. The difficulties in recovering the RT mainly in the 10- and 20-genes concatenates revealed that some incongruence was still present in the 'essential' core. The analysis of the set of genes present in the 'universal' core and not included in the 'essential' core reveals that a substantial portion of the non-vertical signal that differentiates these two core sets is found in this 67-genes subset, which we have referred to as 'non-essential' core.

Therefore, we have shown that essentiality, defined as the intersection between universality and minimal gene set, is a more important factor than universality to recover the vertical signal of proteobacterial genomes. However, we have also shown that the presence of incongruence is not always buffered even in cases where the number of concatenated genes is high. In consequence, we have analyzed the importance of a third factor, namely the function of the genes included in each data set. Due to the nature of the three data sets we have been able to use both supertree and supermatrix approaches. Obviously, the composition of the core is clearly influenced by the special gene composition of the endosymbionts included in the study. These genomes have retained only those genes useful to their symbiotic association and to maintain the essential functions of the cell [27].

Many studies have shown a relationship between gene function and the evolutionary signal encoded therein, associating a higher frequency of lateral gene transfer to operational genes [28,33,34]. We have analyzed this signal in a phylogenomic context taking into account not only the functional category of the genes but also their assignation to each of the three data sets defined previously. In agreement with the results obtained in previous works, the informational categories seem to retain a better vertical signal than operational ones. The supertrees obtained for each of the three data sets with genes in the information category recovered the RT, whereas cellular, metabolism and poorly characterized genes showed a poor performance. In addition, the mean topological distance of each category to the RT confirms the high efficiency of the informational category with respect to the others, whose distance to the RT is significantly higher. However, a more detailed analysis reveals a more complex pattern.

Focusing in the three informational categories, the 'transcription' (K) category recovers the RT in all cases. Furthermore, this is the only category for which supertrees and concatenates perform equally well. Meanwhile the 'Translation, ribosomal structure and biogenesis' (J) category also presents a good efficiency in the concatenate analysis. However, the 'DNA replication, recombination and repair' (L) category only recovers the RT in the 'universal' data set. Therefore, it seems that the 'Transcription' category is a good marker for phylogenomic exploration studies in which the vertical descent relationships of the species have to be assessed.

Metabolism genes usually represent the category with a higher frequency of horizontal gene transfer events [34]. Our analysis corroborates this result, as we have shown that the specialized categories encompassed by this general class have the higher distance to the RT. This result contrasts with the good performance of cellular categories, notably the 'Posttranslational modification, protein turnover, chaperones' (O) and 'Cell motility and secretion' (N) categories. In fact, the relative frequency of these categories is maintained or even increased over the three data sets analyzed. Even more interesting is the case of the 'poorly characterized' genes. Particularly, the 'General function' (R) behaves surprisingly well. Contrary to the 'Function unknown' (S) category, which practically disappears in the 'universal' and 'essential' cores, around 15 genes of the R category are present in these two data sets. The importance of these genes is being recognized now and their influence on bacterial evolution and adaptation is being studied [26,35]. Our results confirm the importance of some of these genes that seem to encompass a good vertical phylogenetic signal.

Finally, it is also remarkable the frequency of RT rejection through the SH test of genes belonging to each functional category. Taking into account the whole genome, around 30% of the gene trees reject the RT and a similar fraction is maintained in the 'universal' and 'essential' cores. This incongruence could be due to the presence of non-vertical signals or to phylogenetic noise (for instance, insufficient signal in the corresponding multiple alignments). The same analysis but splitting the data set by functional category reveals that only the 'Transcription' (K) category has a significantly lower rate of rejection. This means that non-vertical processes and the presence of phylogenetic noise pervade all categories although, as we have shown, genes in some categories are better vertical markers than those in others.

We acknowledge the possible effects that including endosymbiont genomes could have in the recovered phylogenies. The evolution of endosymbiotic genomes is directly influenced by their lifestyle. Due to their relationship with the host, those genes that are not necessary for their survival are difficult to retain. This means that genes related to a free-living style or those related to motility are lost and most of the remaining ones are under weak selection or even in pseudogenization process [36]. This process of genome erosion translates most of the times into high A+T content and substitution rates that, from a phylogenomic point of view, imply possible convergences in the same clade of unrelated genomes, a phenomenon known as "long branch attraction" [37-39]. These features have posed a challenge to traditional phylogenetic methods and are being revealed also as a conflicting point in genome phylogenies, mostly in those based on gene content. Our reference tree assumes the monophyly of the five endosymbionts studied, a result derived in previous works although with some conflicting results [30,31,40-42]. The inclusion in the data set of these genomes has two opposing effects. On the one hand, it reduces the number of genes shared among the species and thus affects the concatenate analyses. However, the number of genes shared by these Proteobacteria excluding these genomes is around 290, not much higher than the 200 genes found here [43]. On the other hand, testing phylogenomic methods with these special conditionings also allows for testing their robustness and more general applicability.

## Conclusion

Phylogenomic analyses are allowing us to study the genome evolution of microorganisms in an extent and detail impossible before the genomic revolution [44]. In the case of evolutionary genomics, current efforts are focusing on the identification of all the evolutionary signals encoded in their genomes. Here we have presented a detailed study on where the vertical signal in Proteobacteria genomes resides. From a phylogenomic perspective, we have shown that the division between informational and operational genes is not as important as previously postulated and that the essentiality of the genes plays an important role in the phylogenetic signal they carry. We have also shown that those sequences classified as 'poorly characterized' are important from an evolutionary perspective as revealed by the gene and phylogenomic trees derived from them and represent a challenge to interpret the evolution of the gene composition of bacteria.

## Methods

### Selection of putative orthologs and definition of cores

We have used the complete genomes of 21 Proteobacteria species (Table 1), including three β-Proteobacteria, one α-Proteobacteria and five endosymbiont genomes belonging to the γ-Proteobacteria group. In a previous work (Comas et al. submitted) we obtained the complete phylome of one of these endosymbionts, *Blochmannia floridanus *[31]. The first step consisted in retrieving the putative orthologs of each protein coding gene in the *B. floridanus *genome. For this, we started by constructing a reference tree with orthologs for 60 informational genes present in all the genomes considered. This reference tree was obtained with the same procedure described in detail below and it represents an expanded version of the tree reported in Gil et al. [31] with additional sequences from the non-γ-Proteobacteria genomes. With this reference tree, we assigned each genome to one of nine different groups (see Figure 1) in order to reduce the BLAST database and to speed up and refine the searches.

Each of the 579 *B. floridanus *protein coding genes was queried [45] against the members of each group. The best hit within a group was used in a subsequent BLAST search against the remaining genomes in that group to retrieve the remaining homologs. We used a minimum threshold of E-value < 1E-03 to consider hits for further analysis. This procedure allowed us to amplify the strength of the searched signal. Most of the genes retrieved in this step were unambiguously putative orthologs. However, we considered that a more stringent test of orthology was necessary before proceeding with the analysis. Hence, we aligned the homologs resulting from the BLAST search for the 579 *Blochmannia *genes and obtained the corresponding maximum likelihood gene trees. Then each homolog was considered as a putative ortholog of the corresponding *B. floridanus *gene once it successfully passed a filtering process based on the following criteria: BLAST report (we used a minimum threshold of E-value < 1E-03), associated functional annotation, length of the alignment, observed and expected position in the gene tree, and adscription to the clusters specified in the Microbial Genome Database for Comparative Analysis [46]. When more than one gene in a genome were identified fulfilling these criteria, we kept the one with best alignment and least likelihood of being a non-orthologous paralog or xenolog for further analysis. Since the *B. floridanus *genome contains no duplicated genes, only one gene per genome was considered in all the analyses. From the 579 alignments, we defined three data sets with different genomic, evolutionary and phylogenetic meaning:

- '*Blochmannia*' core: composed by the 579 annotated protein coding genes of *Blochmannia floridanus *and their corresponding homologs in the other 20 genomes. In this set we deal with from genes present in the 21 genomes to genes present in only four.

- 'Universal' core: the 200 genes of *Blochmannia floridanus *that are also present in the remaining 20 genomes. This set represents those ubiquitous genes in this particular set of genomes but it does not mean that they are essential for bacterial cell life. In this set a fraction of true orthologs and xenologs/paralogs coexist.

- 'Essential' core: from the 200 genes of the 'universal' core we obtained those genes coincident with the proposal for the minimal genome by Gil et al. [27]. This paper describes the 206 genes needed by a cell for a self-sustainable life. From them, 133 genes were present in our 'universal' core and were selected for the 'essential' core and considered as a subset of genes with higher fraction of true orthologs and with essentiality as their common property.

Each data set is composed of the single gene alignments and their derived gene trees. Multiple alignments were obtained with CLUSTALW [47] and later trimmed of positions of ambiguous homology using GBLOCKS [48] with default settings. All the gene trees were inferred using PHYML [49] whose maximum likelihood reconstructions are based on the simultaneous optimization of the topology and branch lengths. In all cases we used the JTT [50] model of amino acid substitution with frequencies estimated from the data set. The proportion of invariant sites was also estimated and we assumed eight discrete rate categories to approximate a gamma distribution for substitution rate heterogeneity among sites.

The congruence of each gene tree was tested against this reference tree by means of the Shimodaira-Hasegawa (SH) test [51] of topologies implemented in TREE-PUZZLE [52].

Genes from each data set were assigned to different functional categories following their annotation in the *Blochmannia floridanus *genome. We used 18 specific functional categories and 4 general ones as defined in the COG database [53].

### Supermatrix analysis

**Figure 5:**
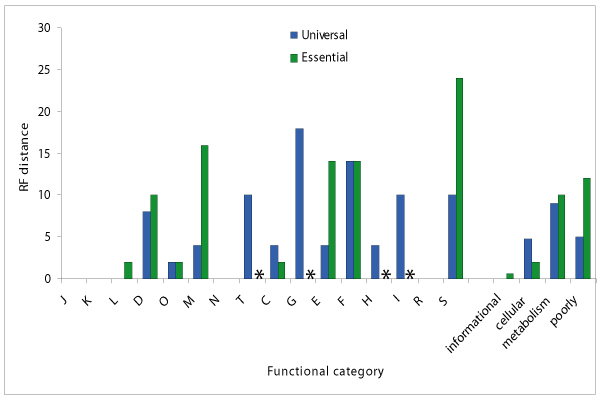
**Reference Tree (RT) and taxonomical classification of the 21 Proteobacteria genomes studied**. The figure is adapted from a previous work (Comas et al. submitted). The taxa and their corresponding taxonomic classification are shown. The topology was obtained in an extensive phylogenomic analysis with the same data set used here. The analyses included a 200-gene supermatrix, a 579-gene supermatrix with coding for absent genes and the supertree derived from the phylome of *Blochmannia floridanus*. Further analyses aimed to correct for the possible influence of GC content bias affecting mainly endosymbiont genomes resulted in the same topology shown here. The branch lengths provided were retrieved from a concatenation of 60 informational genes analyzed by maximum likelihood.

We first analyzed the performance of the concatenate analysis without taking into account the functional assignment of the genes. We carried out two different analyses, one for the 'essential' core and the other for the 'universal' core. One hundred concatenates of 10, 20, 30, 40, 50 and 60 genes were generated randomly from the pool of genes belonging to both core sets resulting in 600 concatenates for each data set. Each one of the 1200 concatenates was analyzed by maximum likelihood using PHYML under the JTT model of evolution and four gamma categories. The computational load prevented us from using more parameters in the evolutionary model. We compared the phylogeny derived from each concatenate with the reference tree shown in Figure 5 by using the Robinson-Foulds distance [54]. This metric measures the number of partitions not shared between two phylogenies and is implemented in the program TREEDIST of the PHYLIP package [55].

### Concatenate and supertree analyses of functional categories

We divided the genes in each core into 18 specific functional and four general categories. For the phylome set of genes, we screened the phylogenetic signal contained in each functional category by obtaining the supertrees derived from the gene trees of each alignment. Differences in the number of species represented in each gene alignment prevented us from performing a concatenate analysis of the whole phylome. However, for the 'universal' and 'essential' cores we were able to obtain the supertree and the concatenate alignments for each functional category.

All the supertrees were obtained with the CLANN software [56]. We employed the commonly used Matrix Representation using Parsimony – MRP [57,58] method. In this method each node of the source trees is coded as a character and a binomial code is assigned to the presence (1) or absence (0) of each taxon in the clade defined by the node. The resulting matrix is analyzed by parsimony. In some cases, the analyses resulted in more than one possible supertree in which case we took into account whether the RT topology was among the most parsimonious topologies found. With the concatenate alignments we obtained the maximum likelihood topology through PHYML [59]. For all the alignments, we used the JTT model of evolution, frequencies estimated from the data set, an estimated proportion of invariant sites and eight gamma rate categories.

Once a supertree and a concatenate phylogeny were obtained for each functional category and core set, we analyzed their phylogenetic signal through their comparison with the RT. The Robinson-Foulds distance as implemented in the program TREEDIST of the PHYLIP package was used to measure the similarity between the obtained topologies and the RT topology. The Shimodaira-Hasegawa test obtained as explained above was also used taking into account the functional assignment of the genes.

## Abbreviations

RT – Reference Tree

RF – Robinson-Foulds

SH – Shimodaira-Hasegawa

COG – Clusters of Orthologous Groups

## Authors' contributions

IC contributed to the conception and design of this study, performed all the analyses and wrote the first draft of the manuscript. AM contributed to the discussion of this work and to the final reviewing of the manuscript. FGC contributed to the conception and design of this study and to the writing, reviewing, and final approval of this article. All authors read and approved the final manuscript.

## Figures and Tables

**Table 1:** List of genomes, with accession numbers and taxonomic assignment, used in this study.

Species (Strain)	Accession No.	Proteobacteria division
*Rickettsia prowazekii*	NC_000963	α
*Neisseria meningitidis *MC58	NC_003112	β
*Neisseria meningitidis *Z2491	NC_003116	β
*Ralstonia solanacearum*	NC_003295	β
*Blochmannia floridanus*	NC_005061	γ
*Buchnera aphidicola *(*Baizongia pistaciae*)	NC_004545	γ
*Buchnera aphidicola *(*Schizaphis graminum*)	NC_004061	γ
*Buchnera aphidicola *(*Acyrthosiphon pisum*)	NC_002528	γ
*Escherichia coli *K12	NC_000913	γ
*Escherichia coli *O157:H7 EDL933	NC_002655	γ
*Haemophilus influenzae *Rd	NC_000907	γ
*Pasteurella multocida*	NC_002663	γ
*Pseudomonas aeruginosa*	NC_002516	γ
*Salmonella enterica *subsp. *enterica *serovar *Typhi*	NC_003198	γ
*Salmonella typhimurium *LT2	NC_003197	γ
*Vibrio cholerae*	NC_002505	γ
*Wigglesworthia glossinidia brevipalpis*	NC_004344	γ
*Xanthomonas axonopodis *pv. *citri *str. 306	NC_003919	γ
*Xanthomonas campestris *pv. *campestris *str. ATCC 33913	NC_003902	γ
*Xylella fastidiosa*	NC_002488	γ
*Yersinia pestis *KIM	NC_004088	γ

**Table 2:** Percentage of gene trees that reject the reference tree using the SH test.

**COG CATEGORY**		***'Blochmannia' *core**	**'Universal' core**	**'Essential' core**	**'Non-essential' core**
Whole data set		29,5	29	27	34,3
**INFORMATION**					
Translation	J	31,5	26,7	26,7	20
Transcription	K	13,3	0	0	0
DNA replication	L	25,8	14,3	16,7	0 *
**CELLULAR**					
Cell division	D	18,2	33,3	100 *	0 *
Posttranslational modification	O	31	33,3	36,4	40
Cell envelope biogenesis	M	34,1	35,7	100 *	25
Cell motility and secretion	N	30	25	20	33,3
Inorganic ion transport and metabolism	P	31,6	NA	NA	NA
Signal transduction mechanisms	T	16,7	NA	NA	50 *
**METABOLISM**					
Energy production and conversion	C	22,7	20	16,7	33,3
Carbohydrate transport and metabolism	G	17,2	29 *	NA	0 *
Amino acid transport and metabolism	E	28	77,8	100 *	75
Nucleotide transport and metabolism	F	33	28,6	50 *	25
Coenzyme metabolism	H	27,3	29 *	NA	50 *
Lipid metabolism	I	36,4	29	NA	50
Secondary metabolism	Q	NA	NA	NA	NA
**POORLY CHARACTERIZED**					
General function prediction only	R	37,8	33,3	37,5	0
Function unknown	S	37,9	29 *	0 *	100 *

Each combination of core-functional category and the different cores proportions are shown. Each cell shows the percentage of cases in which the reference tree topology was rejected at the 0.05 significance level. NA = No data available for this category. Asterisks indicate classes for which only 1 or 2 genes could be used in the analysis.
